# Supplementary material for: Sustainable elk harvests in Alberta with increasing predator populations
Source: PLoS One. 2022 Oct 26;17(10):e0269407. doi: 10.1371/journal.pone.0269407 (PMC9604012; doi:10.1371/journal.pone.0269407)
Supplement: Attachment — Submitted filename: Responses to reviewer comments.docx [file pone.0269407.s001.docx]

Responses to reviewer comments

*Academic Editor*

We agree with your comments and as explained in our cover letter we have dealt with each issue. In particular we have brought on 2 additional coauthors who are the leading experts on large carnivores in Alberta. They have added additional material to describe the increase in predators.

*Reviewer #1*

Thanks for the opportunity to review this manuscript, titled "Sustainable elk harvests in Alberta with increasing predator populations". The ms examines change in estimated elk harvest over 21 years and argues that an increasing trend in estimated harvest indicates that increasing predator populations have not generally impacted elk harvest and that the elk harvest was sustainable during the study period. It's an interesting and very useful topic that should have clear management implications.

At present, there is insufficient information in the ms to assess the reliability of these arguments. Perhaps the strongest inference that could be made from the data as presented is that they don't conform to expectations under the hypothesis that elk populations have been decreasing since 1995. The time series of harvest data should be useful for understanding changes in absolute harvest over time, but more information is needed before it can be said to support the specific hypotheses put forward here.

The main shortcomings that I see at the moment are:

- Harvest estimates are treated as though they are absolute facts that are known without error. However, the extrapolation of reported harvest to a broader hunter population means that there is uncertainty in the estimates, and the voluntary nature of the harvest return data means there could be important nonresponse bias.

We have now addressed the possibility of sampling bias associated with extrapolating from hunter surveys and we reviewed our assumptions with Rob Corrigan who was in charge of the extrapolations to the broader hunter population. As we now note in the manuscript, such a bias might exist but so long as it has not changed over time this will not affect trend detection.

- The trends in harvest, hunter success and effort are all reported without any estimate of uncertainty or precision. Consequently, it is impossible to determine whether the apparent trends are in fact real (i.e. different from zero) given the uncertainty in the data and the model.

This is why we used “route regression” that treats trend estimates as replicates by WMU. This replication clearly showed that the trend is non-zero. We refer the reviewer to the papers that we cited on this method and we insist that our application of this method was correct and appropriate. We disagree strongly with the reviewer’s statement that it is impossible to determine whether the trends are in fact real. This is precisely what we have done. And contrary to the reviewer’s comment we provide measures of precision in Table 3.

- Despite the prominence of predator populations in the title and the abstract, no predator population data are presented or analysed in the manuscript. Hence, any discussion of predator populations is really a side issue rather than a discussion of this study's results.

We recruited one of the world’s leading cougar biologists, Kyle Knopff, and an expert on both wolves and grizzly bears, Andrea Morehouse, to be coauthors to strengthen our treatment of predator populations. These 2 coauthors have published extensively on the 3 primary predators.

All of these problems might be overcome with additional information, but I think it would take a substantial revision.

We have indeed prepared a substantial revision, added 2 more years of data, and we believe that the paper has been substantially improved with this revision.

Detailed comments:

L37: The statement that the harvest estimates indicate that populations have been increasing is tenuous because the precision and reliability of the harvest estimates is unclear, the precision and strength of the relationship between harvest and time is unclear and the relationship between harvest estimates and population density has not been established.

This is incorrect as we noted above.

Introduction

------------

L93: There is no analysis of the effects of predator populations on harvest in the methods or results.

We have expanded our presentation and discussion of predator populations as requested.

L95: There is no comparison of harvest statistics with population estimates in the methods or results.

We note that population estimates are not conducted in a reliable fashion, and for predators these are simply not possible given known methods. But harvest data, hunter success, and hunter effort are the best we can do.

Methods

---------

L126: Different survey methods are prone to different biases (Lukacs et al. 2011). The interpretation of the results should consider the possibility of a shift in bias after survey methods were changed.

Good point, and we clearly indicate when changes were implemented and we also identify the assumptions made by Alberta Environment and Parks in their estimates of harvest. No study of such bias has been conducted.

L129: The assumption that there is no nonresponse bias in voluntary surveys requires a big leap of faith. Do you have any information to help readers make that leap? You should also report the non-response rates here so that we get a feel for how big an issue it may or may not be.

L131: How was the error in the extrapolation process estimated? (e.g. Moloney and Powell 2019)

Same response as above. Indeed, these data assume that extrapolation can be done but so long as this bias remains the same it should not influence our results.

L141: How is success defined here? Is it the number or proportion of successful hunts, or something else?

We clearly defined hunter success as the proportion of hunters who killed an elk.

L144-148: This section is unclear and needs more detail. It seems you've used linear regression, Spearman rank correlation and 'a method similar to route regression' to describe trends in hunter harvest and success over time. The reason for using all of these methods isn't clear. I understand the point of using something similar to route regression to deal with spatial autocorrelation, but you need to provide more detail on how you implemented the models. For example, how were the trends for each management unit weighted? Might a mixed effects model with fixed effect for time and random intercept and slope for management units might be a simpler method for getting an overall trend that accounts for autocorrelation within management units?

We disagree and believe that the reviewer does not understand route regression and has not read the primary papers that we cited. Route regression accounts for temporal autocorrelations not spatial autocorrelation. And the replication is spatial, i.e., by WMU. This is not a mixed model as suggested by this reviewer and the temporal autocorrelation problem certainly would not be resolved by going to a mixed model.

Results

-------

I have answered "no" to review question 2 ("Has the statistical analysis been performed appropriately and rigorously?"). However, this is only because more information is needed in the methods and results, not because the analyses are fundamentally flawed.

No, again this reviewer has not reviewed route regression. We cite the relevant papers.

L171: Rather than reporting the Spearman rank coefficient to describe the strength of the relationship, wouldn't it be informative and internally consistent to just report the R2 from the regression?

No, again this reviewer does not understand route regression. It is the betas of the slopes that vary from WMU to WMU and one cannot rely on r^2^ to resolve the autocorrelation issue.

L172: You should report N and P here with the correlation coefficients. Also later in the results section where you report success statistics.

This is not where the statistics are presented, and to do so would be incorrect. The reviewer does not understand our application of route regression. We have done this correctly.

L190: Your definition of effort needs clarification. Usually, effort would be expressed as number of active hunting days or something similar, but the y axis of Figure 4 suggests that your definition of effort is actually standardised harvest or catch-per-unit-effort (CPUE). If this is the case, then your later assertion that effort has remained constant (L213-217) must be incorrect. If harvest has increased and the ratio of harvest to effort (CPUE) has remained constant, then effort must also have increased.

Ditto from above

Figures 2, 3, 4: You should provide some sort of indication of precision (e.g. error bars) for the harvest, success and effort estimates. These figures are extrapolated from voluntary surveys so the error in the estimation process should be shown. For Figures 2 and 3, the intercept, slope and R^2^ estimates should be reported in the text, along with standard errors or confidence intervals for the intercepts and slopes. The regression lines should also have an indicator of precision.

Ditto from above

I didn't see any mention of where the data were deposited in a publicly available repository. I understand that PLoS1 requires that data are publicly available, and even if this were not the case, I'd strongly recommend it for this study.

Previously, Alberta Environment and Parks indicated that they owned the data. But they have relaxed this stance and now offer the data on line. To follow PLoS ONE guidelines we will publish the data on Dataverse through the University of Alberta library and these data will receive a doi.

Discussion

----------

In general, I think the discussion would benefit from greater clarity of purpose. At present, much of the discussion is speculative, not related to the aims of the ms, or discusses results that don't seem to have a great deal of meaning (e.g. much of the discussion around Table 3). I haven't provided many detailed comments on the discussion because I think it needs a major revision which would make most of my comments irrelevant.

We have indeed conducted a major revision and hope that our coauthorship with Knopff and Morehouse has resolved these concerns.

L205: This seems like a odd way to open your discussion unless this ms is supposed to be a direct rebuttal to Artelle et al. If it is, then this should be made clearer in the introduction.

We are not rebutting Artelle et al. but demonstrating that indeed agencies have not analyzed their data! We now state this explicitly. The first line of the Discussion reads “Although AEP has not evaluated how elk hunter harvest and hunter success has changed in recent years. . .”

L213-217: So a near doubling of general licences and a six-fold increase in limited quota licences had no appreciable impact on total hunting effort? This is surprising and deserves further discussion if it is correct.

Indeed, the number of licensed hunters increased and the hunter success increased. We cannot imagine how this can happen without an increase in the elk population which is the point of this. Effort per hunter has not increased thereby bolstering our interpretation. See lines 260-262 in the revised manuscript.

Table 3, L242: These elements should be presented in the results section. Also, few of the slopes in Table 3 appear to show any strong signal of increase or decrease over time, given the large standard errors relative to the means. In the general harvest section, for example, zone 3 is the only zone in which the confidence interval for the slope seem likely to exclude zero. It's therefore difficult to say that mean harvest increased or decreased in any zone except zone 3 or the limited quota harvest at zone 5.

Ditto from above. This attempt to interpret trends irrespective of the replication by WMU cannot be defended.

Conclusions

-----------

L276: You state that increasing abundance of elk indicates that elk are being managed sustainably, but the ms hasn't presented any elk abundance data, except to note that infrequent aerial survey results exist. You should provide whatever aerial survey results are available somewhere in this ms if you're going to use them to support your assertions.

As noted above, the combination of increased harvest, increased hunter success but no change in effort can only happen with an increase in the abundance of elk. Aerial surveys are conducted about once every 10 years, and for many of the WMUs no aerial surveys have been conducted.

L285-287: Which changes have been linked to increasing predator populations: declining caribou, increasing industrial development, increasing elk, moose and deer?

See lines 320-328 where we address this explicitly.

Lukacs, P. M., Gude, J. A., Russell, R. E. and Ackerman, B. B. (2011) Evaluating cost‐efficiency and accuracy of hunter harvest survey designs. Wildlife Society Bulletin 35(4), 430-437.

Moloney, P. D. and Powell, Z. (2019) Estimates of the 2018 deer harvest in Victoria: Results from surveys of Victorian Game Licence holders in 2018. Arthur Rylah Institute for Environmental Research, Department of Environment, Land, Water and Planning, Heidelberg, Victoria. Available at: https://www.gma.vic.gov.au/__data/assets/pdf_file/0020/503165/Estimates-of-the-2018-deer-harvest-in-Victoria-August-2018.pdf

*Reviewer #2*

Worth publishing – there are many people concerned with long-term trends in harvest rates. This paper provides reasonable evidence that harvest rates are sustainable in some places of Alberta. However, the framing and light/casual treatment of mechanisms needs to be resolved.

Thank you.

Major comments:

The authors have over-sold the ‘carnivore competition hypothesis’ in the framing of this study. There are a number of reasons to expect a decline in harvest and increasing predation from wild carnivores is just one of many plausible explanations. The authors do not test the ‘carnivore competition hypothesis’, nor competing hypothesis, so this is not a reasonable framing of the study as currently written.

We are uncertain how to respond to this but hope that our recasting has helped. Yes, it is very common that hunters and agency personnel believe that large carnivores compete with hunters but these data appear in contradiction.

Second, the statistical analysis is not well described to the point where I am unclear if the correct analytical methods were used. Better explanation is warranted, at minimum. However, I think there are better analyses available for these data.

We think that the method is quite simple (read carefully 161-163), although not widely used outside those analysing BBS and similar trend data. And we disagree that there are better analyses for dealing with temporal autocorrelation. Barry Noon and John Sauer developed the method, and it has seen several reviews. Minor variations exist, but all using the same idea of spatial replication of time series. If the reviewer knows a better analysis he/she should have stated it because we believe that this is wrong.

Minor comments [line #]:

We have corrected all of the errors detected by this reviewer and thank him/her for these details.

[48] the cost of what is high?

[51] define ‘sufficiently’ in an objective manner – what does this mean?

[53] clarify sustainable. Harvest can be sustainable for record bulls, but most managers are attempting to maximize hunter opportunity in a sustainable manner. Eg see your line 64 “…maximize hunter yields…”

[57] remove a repeating word ‘harvests’

[60] there are only 3-point what?

[72] complaints from hunters about lack of access to game comes in many forms, including the issue of increasing competition from wild carnivores. However, many hunters and other conservationists recognize that habitat condition (eg forage availability), weather, disease, roadkill, and competition with other ungulates (cattle, white-tailed deer), or too many hunters - all of which affect hunter success. I appreciate the context behind why the authors want to draw attention to the carnivore expansion hypothesis, but it is one of many potential drivers that also require attention here. Please include these other sources of variation and hypotheses/user-group speculation

[92] define sustainable objectively.

[93] “have elk harvests declined as a consequence of increasing large predator populations?” unless you are measure predator abundance, kill rates, and cause-specific mortality of prey (elk), this objective cannot be answered with the data you have presented here. Safer to ask if elk harvest has declined (period) and then challenge this finding with the hypotheses I suggested above using data you have on hand.

[96] no data on carnivore trends are presented here – again, the trend is a valid hypothesis but the mechanism was not tested. There is no control, for example. You would at least want to compare hunter success in areas with a more stable predator population – such as adjacent to the mountain parks? In which case you would predict that the harvest decline would be steeper in areas where predators are relatively new vs areas where they have been established for most of the study.

[144-148] these methods are unclear. Ye s- there will be temporal autocorrelation and this was not addressed. By doing so, the p-values are inflated because some of these degrees of freedom are not independent. Also, it would make more sense to analyse these with a mixed effects model using WMU as the random effect. I would also consider using a GAM (or GAMM) to relax assumptions of linearity. Were other assumptions of the linear model evaluated (eg distribution of residuals etc)? Why was a liner and Spearman test used – why not use the same approach for both analyses? Further justification is needed.

This is incorrect. See lines 161-163 that details exactly how route regression works, and this eliminates the issues with temporal autocorrelation—guaranteed. A GAM is NOT a solution to temporal autocorrelation, only for nonlinearily.

[169] given the data presented, increasing harvest could suggest an ‘unsustainable’ harvest as well. This is why you need to define sustainable in the introduction. Increasing harvest means more elk are dying by hunters – that’s it. Unless you have data on % of the population harvested (showing that its stable or declining), and that the elk population size is stable or increasing, then I don’t see how you can say this is a sustainable hunt from harvest data alone. Anecdotally, I doubt the elk harvest in Alberta is unsustainable, but the data and arguments presented by the authors are equivocal on sustainability of the harvest. Consider north Atlantic cod fisheries – harvest rates going up or down, on their own, have little to tell use about sustainability.

We have inserted language to define sustainability in context of sustained harvests and populations.

[189] excellent – that hunter effort is stable is key. Perhaps bring this point forward in the results, as the conclusion you mentioned on Line 169 hinge on CPUE (as an index of population size). If this is the basis for the argument of sustainability, then greater effort should be placed on exploring harvest data as an index of population size (see fisheries science for more). This would be a far better use of word space that the carnivore-effect speculation.

[205] – the challenge to the Artelle paper (while worth exploring) seems oddly out of place here. I suggest that you focus on your results and their significance, and then discuss the implications of your findings in the context of the broader literature. There has been much written on why the NA model is successful and there have been many critiques about it as well, including from Artelle et al. Reading this sentence here, the first one of your discussion, would be expected in a rebuttal letter to the Artelle et al paper. The introduction did not establish this challenge as a motivation for the study - so either set this up in the intro or remove it here in the discussion. Finally, by discussing the causality of carnivores in the potential decline of ungulates, without showing data on this or testing it formally, the authors are merely feeding into the narrative of the Artelle paper that they (and others) clearly find issues with. I would take this opportunity to celebrate the strengths of your data and analyses, rather than look to undermine the thesis of one paper written on a very complex issue.

Perhaps we had it wrong in the original draft, but we are not challenging Artelle et al. The first sentence of the Discussion in our revised ms exactly makes the main point of Artelle et al. that agencies do not use the data that they collect!

Table 3: Please indicate which of these Zones are “ mountain units” or “near agricultural areas” with a by adding another column

Done as requested

[248-263] why not test these ideas formally? The variation in slopes in Table 3 could be explained by road density, topography, etc – all of which are readily available through publicly accessible data served such as ABMI.

[287-293] where do elk and caribou overlap?

Through the Peace Country, for example, and certainly in the Willmore Wilderness and Jasper National Park. The last caribou in Banff died in an avalanche.

[217] – there are no data presented on the aerial surveys or their link to the harvest data. Why do the authors keep referring to this information and not showing it?

Redundant with reviewer 1 comment and response

*Reviewer #3*: I reviewed the manuscript “Sustainable elk harvests in Alberta with increasing predator populations” submitted to Plos ONE. This is an interesting compilation and analysis of elk hunter harvest data that is being used to ask whether hunter harvest has increased or decreased in the province of Alberta over a 22 year period. The authors use this data to make the claim that hunter harvest has increased despite the fact that large predator populations have also increased. Below are my general comments as a reviewer of this manuscript. Specific comments and comments that support my general points below are found on the attached pdf with highlights and comments.

Thank you for the supportive remarks.

General comments:

1) Overall I think this is an interesting and likely trustworthy analysis of the impact of large carnivores on hunter harvest of elk. I qualify this as “likely” primarily because the authors do not include any information on the distribution and abundance of predators and this is very important to this paper. Without this type of data, it is impossible to determine whether their analyses are correct or not. For example, the authors show that elk harvest has declined in zone 4 (mountaneous area) and claim that this is due to grizzly bear predation. But we have no idea whether zone 4 is the only unit that has grizzly bears. If zone 4 is the only unit with grizzly bears then their claims need to be adjusted and to say large carnivores don’t impact hunter harvest is false. It is possible that elk populations are growing in other zones without predators and that more hunters are going to these units to hunt and thus accounting for the overall trend in increased hunting in Alberta. Thus I can’t see publishing this article without a fair amount of understanding of the spatial distribution and estimates of abundance of carnivores in each of the 5 zones. It’s very possible you are comparing zones with and without large carnivores.

We concur and in response we recruited Knopff and Morehouse as coauthors thereby resolving nearly all of these concerns.

2) I realize the philosophy of this journal is to focus on the methods and results and not care much about how the article is “packaged”. However, I found the introduction to be very incongruent with the title and abstract and presumably the main point of the article that hunting has not declined with the restoration of large predators. There was very little mention of large carnivore background for readers unfamiliar with the plight of carnivores and the first two paragraphs seemed very out of context and likely could be deleted or at least re-written to better incorporate the theme of the presumed paper.

Yes, again, the lack of data on carnivores was noted by all 3 reviewers.

3) The inherent issues of the temporally correlated data in each of the GMUs is an important topic and the details of how the author’s handled this aspect of the data is very vague. I’m not suggesting that the analyses they performed is inappropriate but I am saying it is difficult if not impossible to evaluate because there is no detail.

We do not believe that it is vague, and it’s actually quite simple as explained in lines 161-163. These methods appear to have been used exclusively by avian ecologists.

4) The data on hunter harvest is based on surveys. I would ask the authors to be more thoughtful of possible inherent issues and limitations of this data. Are there suspected or known biases or known or estimated error rates/ or confidence intervals that can be reported? In the discussion it would be appropriate to qualify your results and inference with acknowledgement of any issues.

Yes, this issue was a concern and we met with Rob Corrigan who compiled these data and discussed possible bias. Indeed, AEP assumes that the hunters who failed to report have harvested elk at the same proportion as for those who did report. This is likely an incorrect assumption—hunters would seem more likely to report if they actually went hunting and did harvest an animal. But so long as that bias is consistent among WMUs and through time, it would not affect the trends that we have documented.

*Reviewer 3 inserted comments in a pdf document*. We have corrected the wording issues that he/she identified there and appreciate the attention to details.
